# Evolving Strategies in the Detection and Management of Left Ventricular Thrombus: A Clinical Summary

**DOI:** 10.2174/011573403X364065250429095440

**Published:** 2025-05-09

**Authors:** Mahmoud Abdelnabi, Ramzi Ibrahim, Hoang Nhat Pham, Yehia Saleh, Abdallah Almaghraby

**Affiliations:** 1 Department of Cardiovascular Medicine, Mayo Clinic, Phoenix, AZ 85054, USA;; 2 Department of Medicine, University of Arizona, Tucson, AZ, 85721, USA;; 3 Division of Cardiology, Montefiore Medical Center, Albert Einstein College of Medicine, Bronx, New York, NY, 10461, USA;; 4 Department of Cardiology, Faculty of Medicine, Alexandria University, Alexandria, Egypt;; 5 Department of Cardiology, Ibrahim Bin Hamad Obaidallah Hospital, Ras Al Khaimah, UAE

**Keywords:** Left ventricular thrombi (LVT), cardiomyopathies, cardiac imaging, anticoagulation, direct oral anticoagulants, thromboembolic events, stroke

## Abstract

Recent advancements have emerged in understanding the epidemiology and optimal therapeutic options for left ventricular thrombi (LVT). With early percutaneous interventions in acute myocardial infarction, the prevalence of LVT has decreased. However, the best strategies for prevention, risk stratification, and management remain unclear, especially among non-ischemic cardiomyopathy disorders. This review outlines these advancements and provides an overview of the diagnostic and therapeutic implications of LVT in ischemic and non-ischemic cardiomyopathies. Significant gaps in the current evidence persist, particularly regarding the optimal timing for LVT screening and the need for prophylactic anticoagulation, highlighting opportunities for prospective cohort studies. Furthermore, a better understanding of the unique risk factors that contribute to increased LVT risk would lead to more comprehensive algorithms that may quantify the risk of LVT development, aiding in developing preventive strategies targeted at reducing rates of LVT. Until more definitive evidence is available, clinicians should custom LVT screening, preventive measures, and management strategies based on individual patient risk factors.

## 1 INTRODUCTION

Left ventricular thrombus (LVT) is a significant clinical condition characterized by thrombi formation within the left ventricle, often due to myocardial injury, dysfunction, or structural abnormalities. The presence of LVT is associated with an increased risk of systemic thromboembolic events, which can cause significant morbidity and mortality in affected patients. LVT is most commonly noted in ischemic heart disease, particularly after large anterior myocardial infarctions, but it can also occur in various other clinical conditions, including non-ischemic cardiomyopathies, myocarditis, and hypercoagulable states [1, 2]. Advances in diagnostic modalities have improved the detection and management of LVT, enhancing the understanding of its pathophysiology and natural history, risk factors for embolic complications, and treatment options [1, 3]. Despite this, the management of LVT remains challenging, with several evidence gaps remaining in the literature, including the type and duration of anticoagulation needed to prevent thromboembolism without increasing the potential risk for bleeding complications. Promising data may suggest a future alternative for LVT treatment [4]. Given the prevalence of LVTs in clinical practice, this review provides a comprehensive overview of LVT, including its epidemiology, pathophysiology, diagnostic approaches, management strategies, and future directions, aiming to provide clinicians with clinical decision-making algorithms based on the current evidence in daily clinical practice.

## 2 EPIDEMIOLOGY

Despite significant advancements in reperfusion strategies and antithrombotic therapies, the incidence of LVT following acute myocardial infarction (AMI) remains substantial [1, 5-12]. Recent data show that within 90 days of an ST-elevation myocardial infarction (STEMI), the incidence of LVT detected by echocardiography was 9.1% in patients with anterior STEMI and 2.7% across all STEMI cases [1]. Similarly, a meta-analysis found that within one month of STEMI, LVT incidence detected by cardiac magnetic resonance (CMR) was 12.2% in anterior STEMI patients and 6.3% in all STEMI patients [13]. This incidence rose significantly to 19.2% in patients with anterior STEMI and a left ventricular ejection fraction (LVEF) below 50%. Additionally, prior studies have suggested that LVT can develop in up to 36% of patients with dilated (non-ischemic) cardiomyopathies [3].

## 3 PATHOGENESIS OF LVT

The pathogenesis of LVT involves a combination of blood stasis due to global reduced ventricular function and/or regional wall motion abnormalities, endocardial injury, and a subsequent inflammatory environment [5]. LVT is associated with up to 22% of cerebral and peripheral arterial thromboembolic events and carries a 37% risk of major adverse cardiovascular events (MACEs). Embolization risk is primarily linked to the mobility and protrusion of the thrombus, while thrombus size plays a lesser role; smaller LVTs are more likely to regress and are associated with a lower risk of morbidity and mortality.

## 4 DIAGNOSIS OF LVT

### 4.1 Imaging Modalities

The optimal timing for imaging to detect left ventricular thrombi (LVT) after acute myocardial infarction (AMI) remains a topic of debate. The risk of LVT formation is highest within the first two weeks following AMI. Studies have shown that imaging performed 1 to 2 weeks after AMI, using transthoracic echocardiogram (TTE) or cardiac magnetic resonance (CMR), reveals a higher LVT incidence than imaging performed in the initial days. Delayed imaging, particularly with contrast echocardiography or CMR, may benefit high-risk patients if anticoagulation therapy is not immediately planned. However, no evidence suggests that repeated imaging can reduce embolic event rates [3, 5, 14].

TTE is often the first-line imaging modality for detecting LVT due to its availability and affordability compared to other techniques. While TTE offers high specificity (95% to 98%), it has relatively low sensitivity (21% to 35%), which may result in occasional missed LVTs due to poor visualization of the left ventricular apex, small thrombi, or suboptimal acoustic windows [3, 5, 14]. Transesophageal echocardiography (TEE) adds little diagnostic value in LVT detection, as the left ventricular apex is often foreshortened and difficult to visualize through transgastric views, particularly in patients with a dilated left ventricle and apical dyskinesis [5]. Contrast echocardiography can enhance TTE sensitivity and specificity, up to 64% and 99%, respectively, by improving the definition of the endocardial border, making it a useful tool in cases where the apex is poorly visualized (class IIb, level of evidence C) [3, 15].

CMR is considered an excellent modality for assessing LVT, offering a sensitivity of 82% to 88% and a specificity close to 100%, compared to surgical or pathological confirmation [3, 15-17]. Its superiority over TTE or contrast echocardiography lies in its higher resolution and the ability to provide detailed tissue characterization using late gadolinium enhancement (LGE). CMR also helps identify structural risk factors for LVT, such as severe left ventricular dysfunction, aneurysms, apical wall motion abnormalities, and high myocardial scar burden or infarct size [3, 5]. However, widespread CMR use is limited by cost and availability. Current guidelines recommend CMR in patients with inconclusive echocardiography results or when clinical suspicion of LVT remains high (class IIa, level of evidence C) [14]. It is worth noting that LVTs detected by CMR, but not by echocardiography, are often smaller and mural in morphology, and there is limited evidence that anticoagulation improves outcomes in these patients, particularly those at high risk for bleeding [3, 5].

Cardiac computed tomography (CT) has demonstrated high sensitivity (100%) and specificity (92%) for detecting left atrial appendage thrombi, with a negative predictive value of 100%. However, its positive predictive value is lower at 23% compared to TEE. However, data on the effectiveness of cardiac CT in detecting LVT are limited to small cohort studies and case reports, and no studies have validated its accuracy against pathology or clinical outcomes [3].

Positron emission tomography (PET) techniques have shown promise in diagnosing LVT. Recent studies have highlighted the utility of PET imaging with novel radiotracers for detecting thrombus formation. For instance, using a glycoprotein IIb/IIIa receptor antagonist-based radiotracer, F-GP1, has demonstrated the ability to noninvasively detect thrombus formation in coronary arteries and other cardiac regions, including the left ventricle [18]. Additionally, hybrid imaging modalities, such as PET/MRI, have shown their potential to provide comprehensive anatomical and functional assessment. A study using a fibrin-binding radiotracer, [Cu]FBP8, combined with PET/MRI, demonstrated excellent accuracy in detecting left atrial appendage thrombi, suggesting potential applicability for left ventricular thrombi as well [19]. This integrated approach offered detailed information on thrombus composition and biological properties, enhancing diagnostic precision.

### 4.2 Biomarkers

Studies have suggested a promising role of circulating biomarkers, such as D-dimer and prothrombotic factors, in the risk stratification of LVT. Elevated D-dimer levels have been associated with an increased risk of LVT formation in various patient populations, such as DCM and post-MI patients. Elevated D-dimer levels (>444 ng/mL DDU) were independently associated with an increased risk of LVT in patients with DCM [20]. Similarly, D-dimer levels were useful in diagnosing and assessing the risk of intracardiac thrombus in DCM patients, with an optimal cut-off value of 484 ng/mL [21]. Additionally, other studies have highlighted the predictive value of D-dimer in post-MI patients. D-dimer was an independent predictor of LVT formation in post-MI patients with left ventricular dysfunction, with an optimal cut-off value of 1.53 mg/L [22]. Similarly, higher fibrinogen levels have been linked to early LVT formation in anterior wall STEMI patients [23]. Elevated levels of high-sensitivity C-reactive protein (hs-CRP) have been independently associated with the presence of LVT in patients with ST-elevation myocardial infarction (STEMI) treated with primary percutaneous coronary intervention (PPCI) [24]. Higher white blood cell count (WBC) and neutrophil-to-lymphocyte ratio (NLR) upon admission have been shown to predict LVT formation in STEMI patients [25]. These findings suggest that different biomarkers may be useful in LVT risk stratification as a marker of inflammation, coagulation, and fibrinolytic system activation; however, their routine use in clinical practice remains questionable and requires further validation through larger, prospective studies.

### 4.3 LVT Prevention and Treatment

**Fig. (1):**
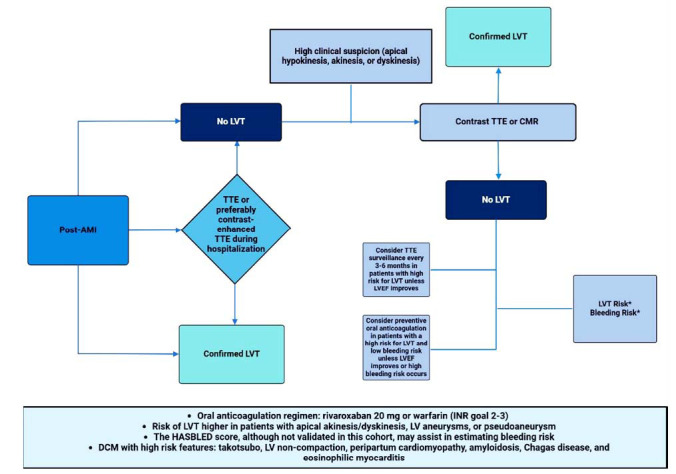
Post-AMI LVT prevention and management. Diagnostic and management algorithm for LVT in AMI.

**Fig. (2):**
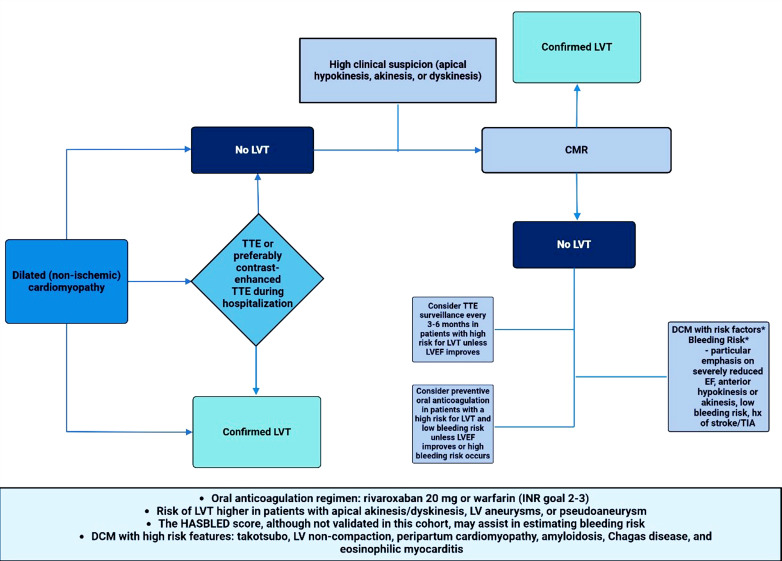
Dilated cardiomyopathy LVT prevention and management. Diagnostic and management algorithm for LVT in dilated cardiomyopathy.

Although evidence supporting the use of anticoagulation for LVT prevention is limited, current recommendations suggest that prophylactic anticoagulation with vitamin K antagonists (VKA) may be considered for patients with STEMI and anterior apical akinesis or dyskinesis (class IIb, level of evidence C) [3, 5] (Fig. **1**). In contrast, data on LVT prevention in non-ischemic cardiomyopathies, with or without additional risk factors, are scarce [26, 27] (Fig. **2**). The European Society of Cardiology (ESC) expert consensus suggests that in patients with Takotsubo syndrome, anticoagulation with intravenous or subcutaneous heparin may be considered in the presence of left ventricular (LV) dysfunction and apical ballooning [9]. Similarly, the Heart Rhythm Society guidelines recommend anticoagulation in patients with LV non-compaction and LV dysfunction, though this is based on limited case series data without a control group (class IIb, level of evidence B-NR) [28, 29]. The AHA/ASA 2021 Guideline for the Prevention of Stroke in Patients With Stroke and Transient Ischemic Attack also supports the use of oral anticoagulation in patients with LV non-compaction who have had a recent transient ischemic attack or ischemic stroke (class IIa, level of evidence C) [30]. In patients with peripartum cardiomyopathy, who are at increased risk of LVT due to the hypercoagulable state associated with pregnancy, the 2016 AHA scientific statement recommends anticoagulation in those with severely reduced LV ejection fraction [31]. The ESC Heart Failure Association also includes this recommendation in cases of acute peripartum cardiomyopathy with LV ejection fraction below 35% [32]. Despite evidence of increased LVT rates in cardiac amyloidosis, chemotherapy-related cardiomyopathy, hypertrophic cardiomyopathy, Chagas cardiomyopathy, and eosinophilic myocarditis, there are no prospective studies or clinical trials to support anticoagulation in these populations for LVT prevention [3, 5, 33-37].

Current guidelines recommend anticoagulation based on retrospective registry data and smaller observational studies to treat established LVT. Patients with dilated cardiomyopathy and an LVT are typically treated for 3 to 6 months with either VKAs or direct oral anticoagulants (DOACs) [38-40]. Treatment may be discontinued earlier if the LV ejection fraction improves above 35% and the LVT resolves or if major bleeding occurs. Conversely, anticoagulation may be extended in cases where LVT persists, or LV systolic function remains impaired, particularly with ongoing apical akinesis or dyskinesis. A retrospective analysis of the No-LVT trial data revealed a 9.61% risk of LVT recurrence at a mean follow-up of 81±26.08 days, with LVEF being the strongest predictor of recurrence following anticoagulation cessation [4]. Another study reported a 24.3% recurrence risk at a median follow-up of 1.2 years, with LV aneurysms identified as an independent risk factor. LVT recurrence was also linked to a higher risk of thromboembolic events [41]. In patients with coexisting hypercoagulable or pro-inflammatory states, such as malignancy or renal failure, prolonged anticoagulation may be necessary. However, the risk of bleeding must be carefully balanced through shared decision-making.

The ACC/AHA 2013 STEMI guidelines recommend a 3-month course of oral anticoagulation for patients with myocardial infarction and mural LVT in conjunction with dual antiplatelet therapy (DAPT) (class IIa, level of evidence C) [5]. Recent studies suggest that DOACs are non-inferior to VKAs, offering faster thrombus resolution and a lower risk of bleeding and thromboembolic events [4]. The updated 2023 ESC acute coronary syndrome (ACS) guidelines recommend oral anticoagulation for 3 to 6 months following LVT diagnosis, with either VKAs or DOACs, guided by follow-up echocardiography or CMR imaging [14]. Given the lack of prospective data on anticoagulation duration, particularly when combined with antiplatelet agents, treatment should be individualized based on the patient’s clinical status, bleeding risk, and follow-up imaging results [14].

### 4.4 LVT Recurrence

Evidence regarding the prevalence, risk factors, and clinical outcomes of LVT recurrence remains limited. A retrospective analysis of the No-LVT trial data showed a 9.61% risk of LVT recurrence at a mean follow-up of 81±26.08 days, with LVEF being the strongest predictor of recurrence after anticoagulation discontinuation [42]. Another retrospective analysis suggested that LVT recurrence risk can be as high as 24.3% at a median follow-up of 1.2 years, with LV aneurysms being an independent risk factor. Furthermore, LVT recurrence was associated with a higher risk of thromboembolic events [41]. However, other studies have reported lower rates. In a retrospective study, patients with a history of LVT had an annual recurrence rate of 2.3% during a follow-up period of approximately 57.5 months [43]. Similarly, a recurrence rate of 5.3% was reported in a cohort of patients using serial CMR over a median follow-up of 255 days [44]. The findings emphasized the significance of extended anticoagulation, particularly for patients with unresolved thrombi at six months or high-risk patients with reduced LVEF or LV aneurysms.

### 4.5 Mechanisms of Action of DOACs in LVT

Generally, DOACs, including factor Xa inhibitors (*e.g.*, rivaroxaban, apixaban, edoxaban) and direct thrombin inhibitors (*e.g.*, dabigatran), exert their anticoagulant effects by targeting specific steps in the coagulation cascade. Factor Xa inhibitors bind directly to factor Xa, a key enzyme responsible for converting prothrombin to thrombin, thereby reducing thrombin generation and subsequent fibrin formation, which inhibits clot formation [45, 46]. Direct thrombin inhibitors directly inhibit thrombin (factor IIa) activity, preventing fibrinogen conversion to fibrin and further preventing thrombin-mediated activation of coagulation factors V, VIII, and XI, which are involved in clot propagation [45, 46]. Meanwhile, in LVT, the proposed mechanisms of DOACs include inhibiting key steps in the coagulation cascade to prevent thrombus formation and promote thrombus resolution. Additionally, some studies suggest that DOACs may enhance fibrinolysis by accelerating tissue plasminogen activator (t-PA)-induced fibrinolysis by increasing plasmin generation, which is dependent on thrombin-activatable fibrinolysis inhibitor (TAFI) [47].

## 5 FUTURE DIRECTIONS

### 5.1 Artificial Intelligence

Artificial intelligence (AI) has shown significant potential in the diagnosis, risk stratification, and treatment of LVT, primarily through cardiac imaging and predictive analytics advancements. AI algorithms, such as machine learning (ML), deep learning (DL), and convolutional neural networks (CNN), have demonstrated high accuracy in the assessment of cardiac function and detection and risk stratification of myocardial abnormalities, such as LVT, through enhanced image acquisition and automated data analysis from various imaging modalities [48]. Moreover, AI has been employed in predictive analytical models to predict the risk of thrombus formation and related complications. By integrating clinical and imaging data, AI models can predict patient outcomes and guide treatment decisions, potentially improving clinical management and patient care [49].

### 5.2 Precision Medicine

Integrating the precision medicine approach and AI algorithms in LVT diagnosis and treatment has significant potential for improving risk stratification, tailoring treatment strategies to individual patient profiles, optimizing clinical outcomes, and minimizing risks [50].

## CONCLUSION

Despite significant advances in reperfusion, pharmacological therapies, and device interventions for both ischemic and non-ischemic cardiomyopathies, the optimal approach to diagnosing and managing LVT, particularly after acute myocardial infarction, remains contentious due to gaps in current evidence [11, 38, 51-55]. Future research should prioritize identifying the ideal timing and imaging modalities for improved LVT detection while also exploring the clinical significance, natural history, and customized anticoagulation strategies based on LVT morphology. The timing of LVT screening, particularly in relation to risk prognostication, remains unclear. Cohort studies may provide valuable insights into key prognostic factors that can enhance risk stratification. It is also crucial to evaluate the risks and benefits of prophylactic anticoagulation in high-risk cardiomyopathy patients, as well as the necessity of indefinite anticoagulation in individuals with persistent or recurrent LVT. High-risk groups, such as patients with hypertrophic obstructive cardiomyopathy and amyloidosis, remain understudied regarding LVT development, limiting the evidence available to guide regular anticoagulation use in these populations. Current guidelines do not differentiate between revascularized and non-revascularized STEMI cases with anterior wall motion abnormalities regarding LVT prevention or treatment strategies [56-58]. Prospective studies could help integrate different biomarkers, multimodality imaging, and AI models, and define the optimal duration of anticoagulation based on revascularization status, ultimately improving risk stratification and preventing LVT development or recurrence. Based on the present data, a decision-making algorithm is proposed for diagnosing and managing LVT in different clinical scenarios. Until more robust evidence is available, clinicians should personalize the screening, monitoring, and treatment of LVT, carefully weighing the individual patients’ risk of thromboembolic events against their risk of bleeding.

## LIST OF ABBREVIATIONS

AMI: Acute Myocardial Infarction
CMR: Cardiac Magnetic Resonance
DOACs: Direct Oral Anticoagulants
LVEF: Left Ventricular Ejection Fraction
LVT: Left Ventricular Thrombi
MACEs: Major Adverse Cardiovascular Events
